# “It's Been Negative for Us Just All the Way Across the Board”: Focus Group Study Exploring Parent Perceptions of Child Screen Time During the COVID-19 Pandemic

**DOI:** 10.2196/29411

**Published:** 2021-06-08

**Authors:** Amber J Hammons, Elizabeth Villegas, Ryan Robart

**Affiliations:** 1 Department of Child and Family Science California State University, Fresno Fresno, CA United States; 2 NORC at the University of Chicago Chicago, IL United States

**Keywords:** children, COVID-19, experiences, family, outcomes, pandemic, parenting, parents, screen time

## Abstract

**Background:**

Child screen time (ST) has soared during the COVID-19 pandemic as lockdowns and restrictions have forced changes to regular family routines. It is important to investigate how families are navigating ST.

**Objective:**

This study aimed to explore families’ experiences of ST during the COVID-19 pandemic.

**Methods:**

Virtual focus group sessions were conducted between December 2020 and February 2021 in English and Spanish. Transcripts were analyzed using reflexive thematic analysis.

**Results:**

In total, 48 parents (predominantly Hispanic) residing in California participated in 1 of 14 focus group sessions. Children were attending school remotely at the time of the study. A total of 6 themes and 1 subtheme were identified: (1) total ST has increased; (2) children are too attached to screens; (3) ST has advantages and disadvantages but parents perceive ST as mostly negative; (4) parents and children have limited options; (5) ST restrictions (subtheme: children react negatively when ST is restricted); and (6) parents are concerned that children are not getting enough exercise.

**Conclusions:**

This study provides a cross-sectional insight into how family life has changed with regard to ST during the COVID-19 pandemic. Parents expressed concerns about total ST, the addictive nature of it, and lack of physical activity. It is important that future studies examine the long-term effects of heavy ST and preemptively introduce ways to redirect children’s ST habits as the country attempts to establish a new normal.

## Introduction

The COVID-19 pandemic has transformed family life in myriad ways. The pandemic has presented an unprecedented situation where for many, work and family life have collided [1,2]. Some parents are finding themselves working from home for the first time [3], and in addition they may also be juggling distance-learning for their children [4]. Concomitant effects of the pandemic include fewer support systems available for assistance, such as in-person schooling [4,5], child care [2,6], and even emotional support [7], thus exacerbating the complexity of the situation for parents and families. The pandemic has forced many families to create new norms and routines to reestablish an equilibrium within their homes and lives.

An area that has been affected within families is screen time (ST) [8,9]. The use of screens has increased for children as many schools nationwide shifted from conducting classes in person to conducting them remotely. Lockdowns and closures of schools, recreational centers, and organized sports have also prevented families from engaging in regular activities. Singularly and collectively, these actions are likely to lead to an increase in the use of screens in households nationwide.

ST is associated with both positive and negative outcomes among youth [10]. Potential benefits include opportunities to engage socially with others [11], including actively engaging on social media (eg, posting content), which may provide some protection against depression [12]. ST has also been linked to children’s learning [13], higher friendship quality, and a positive academic orientation [14]. Included among the negative associations are depression and anxiety [15], lower well-being [15], sleep disturbance [16], weight gain [17], and adverse dietary outcomes [18]. Observational and experimental studies have linked ST to an increased risk of obesity, and increased energy intake has received support as an explanatory mechanism [19]. Snacking during television viewing is associated with an increased risk of obesity [20], which could be exacerbated during times of stress and magnified by preferences for comfort foods. One study found that youth who watch the television for more than 5 hours a day have 5-fold higher odds of being overweight than those who watch the television for less than 2 hours a day [21]. An additional mechanism that has received support in explaining the link between ST and obesity is targeted advertising, which has intensified during the pandemic [22].

The American Academy of Pediatrics (AAP)’s guidelines for media use in school-aged children and adolescents calls for a healthy balance to be established by parents and pediatricians [23]. Parents and pediatricians are encouraged to work together to create a Family Media Use Plan that sets boundaries and is in the best interest of the child, prioritizing sleep, exercise, and breaks from media. Parents may need to reevaluate their family ST practices during a pandemic that causes changes in usual routines.

The objective of the present study is to describe families’ experiences of ST during the COVID-19 pandemic. While it is expected that ST will increase, particularly owing to remote schooling, it is not clear how families are managing overall ST or what parents’ perceptions are of their child’s ST, and how ST is influencing family functioning during this time. Given the unprecedented situation of school closures and cyclical lockdowns, there exists a lacuna of studies on ST in families during the COVID-19 pandemic. As the nation prepares to return to a new normal, it is important to understand how ST within families will evolve as well as to anticipate any long-term attendant effects.

## Methods

### Sample Selection

Parents were eligible to participate in the study if they had at least 1 child between the ages of 5-18 years and access to the internet. Recruitment strategies included inviting parents to participate from a prepandemic contact list of families that had indicated their interest in participating in a health study for Hispanic families (fliers were handed out at grocery stores, flea markets, local schools, and churches). Additionally, participants were recruited by word of mouth, snowballing, social media (eg, an announcement on a Parents’ Group), and an email announcement through a student listserv in the Child and Family Science Department at California State University, Fresno.

### Data Collection and Participants

The present study is part of a qualitative study designed to explore mealtimes, ST, and family functioning during the COVID-19 pandemic. A focus group guide was created to tap into family life during the pandemic and was developed on the basis of previous studies on family functioning and process, as well as a review of the ST literature. Focus group methodology was selected as the study design owing to the unprecedented nature of the pandemic and with the goal of offering a space for parents to engage in an open discussion on family life. In total, 48 parents participated in 1 of 14 focus group sessions (average of 3 participants per group) that were conducted between December 2020 and February 2021. Focus group sessions were conducted in English (n=6) and Spanish (n=8). All focus group sessions were conducted on Zoom and lasted 70 minutes on average. Focus groups were recorded and led by 2 trained facilitators. Participants received a US $25 gift card for participating in the focus groups and filling out an anonymous brief demographics survey that included questions about employment, remote work, essential worker status, virtual schooling, and location.

The Department of Child and Family Science’s Review Committee for the Protection of Human Subjects at California State University, Fresno, approved the study. Participants provided both written and verbal informed consent before participation.

### Demographics of the Study Participants

In total, 48 parents participated in the study. All participating parents were mothers, with the exception of 1 father. Participants resided in California; most (94%) lived in the Central Valley. Our participants were predominantly Hispanic (n=39 of 48, 81%), 7 (15%) identified as White, and 2 (4%) identified as Asian. All children were engaged in distance-learning at the time of the study. The average age of the children was 11.13 (SD 5.87) years and that of the parents was 37.48 (SD 8.37) years. The median number of children per family was 2.5. More than half (n=29 of 48, 60%) of the sample reported an annual income of less than US $50,000, and most were married or cohabitating (n=38 of 48, 79%). In total, 12 of 48 (25%) participants had a bachelor’s or master’s degree. A total of 18 (38%) parents were employed, half of whom (n=9 of 18, 50%) were working from home at the time of the study, and approximately half (n=25 of 48, 52%) of the sample comprised essential workers or lived with one. Table 1 shows a breakdown of the participant characteristics.

**Table 1:** Demographic characteristics of the parents included in this study (N=48).

Characteristics			Values
Parent age (years), median (IQR)			37.5 (32.0-43.8)
Child age (years), median (IQR)			11.5 (7-15)
Children per family, median (IQR)			2.5 (2-4)
**Employment status, n (%)**			
	Employed	18 (38)	
	Working remotely	9 (19)	
**Education, n (%)**			
	Less than high school	14 (29)	
	High school	14 (29)	
	Technical school	4 (8)	
	Professional degree	2 (4)	
	Bachelor’s degree	5 (10)	
	Master’s degree	7 (15)	
	Declined to respond	2 (4)	
**Annual income (US $), n (%)**			
	≤19,999	13 (27)	
	20,000-29,999	5 (10)	
	30,000-39,999	6 (13)	
	40,000-49,999	5 (10)	
	50,000-59,999	3 (6)	
	60,000-69,999	3 (6)	
	70,000-79,999	2 (4)	
	80,000-89,999	1 (2)	
	90,000-99,999	1 (2)	
	≥100,000	8 (17)	
	Declined to respond	1 (2)	

### Data Analysis

Audio recordings were transcribed verbatim by research assistants. The 8 focus group sessions in Spanish were transcribed and translated to English by 3 proficient bilingual research assistants. Back-translations were then performed by a proficient bilingual research assistant who had no exposure to the original recording or transcript. To ensure accuracy in the translations, back-translations were compared to the original transcripts. Discrepancies were minor and resolved through consensus.

Transcripts were analyzed using reflexive thematic analysis [24] and an inductive approach within a phenomenological framework. Three researchers (AJH, EV, and RR) familiarized themselves with the transcripts and created their own codes through data immersion. Researchers shared the codes, agreed on them, and applied them independently to the transcripts. Researchers regularly discussed the codes and revised and refined them as necessary. Patterns of meaning in the data guided the generation of preliminary themes, and the codes were then reexamined by the research team to ensure the fit of the themes. The final step involved selecting representative quotes. Data were analyzed using Dedoose (version 8.3.47, University of California, Los Angeles) [25].

## Results

### Results Overview

Themes did not differ between the parents who participated in the English and Spanish language group sessions. Six key themes and one subtheme were identified: (1) total ST has increased; (2) children are too attached to screens; (3) ST has advantages and disadvantages but parents perceive ST as mostly negative; (4) parents and children have limited options; (5) ST restrictions (subtheme: children react negatively when ST is restricted); and (6) parents are concerned that children are not getting enough exercise. Table 2 contains representative quotes for each theme and subtheme.

**Table 2:** Themes and representative quotes.

Themes	Quoted responses
Theme 1. Total ST^a^ has increased	“Oh, good God, I would have to say that with me, I feel like my kids are on the computer all the time.” [Participant #1, mother] “So, I mean, they’re on it, except for dinnertime, they are on the computer.” [Participant #4, mother] “So, screen time has exponentially increased in the pandemic.” [Participant #16, mother]
Theme 2. Children are too attached to their screens	“All day long, since dawn my daughters are already watching TV, they are with the iPad in hand or with the phone and it is practically all day. If they go out to play, it is for very little, and it is because I have to be behind them, ‘leave me the phone, turn off the TV’. I go and I turn them off. If it was up to them, all day long they will be using their phone or TV.” [Participant #29, mother] “It’s like they want to be there all the time so I literally have to remove the device, put them away where they can’t get them so they can go do something else. So, they definitely struggle with just kind of wanting to do that all the time.” [Participant #14, mother] “Mine too, since the pandemic started. Classes on the computer, the phone, the games...the little girl who is 7 is addicted to the telephone. She has made it difficult to take it away because we don’t go outside much. Yes, even me, because I have a part-time job. Since everything is on social networks, we spend more time on the phone. My husband is the only one not so much on the phone because he has to work. When he arrives [home] he is on his phone.” [Participant #38, mother] “I’ve noticed my kids have that addictiveness too. When they get in trouble, that’s the thing, we take away from them and it’s almost like you are taking, I hate to say it, like drugs away from a drug addict, they just get so upset and so emotional and it’s just, it’s the end of the world for them. I, I don’t like that at all, so it’s kind of like, what are we going to do, it’s because of the situation that we are in, darned if you do, darned if you don’t situation, because they need it for school and they need it for social interactions like, so I feel you almost have no choice in this situation.” [Participant #13, mother]
Theme 3. ST has advantages and disadvantages, but parents perceive ST as mostly negative	“I think the good thing that we've noticed is that both of these kids have become extremely knowledgeable on technology. And so, for that, I would say that's a positive. I can tell you that at four years old, I would not have been able to do the things that they do on technology. But I think that it's good that they're learning these skills and things as young as they are. I just want to make sure that it doesn't continue to be their entire worlds. And they still get those opportunities to do the kids things like go outside and play, and imagine, and get creative outside of technology too.” [Participant #19, mother] “Well, I don’t like the amount of screen time, I don’t think it’s a positive thing overall. That being said, it’s been a way for her to connect with people, with friends and that’s been good for her mental health to have that connection, and that’s why I’ve allowed her to be on her screen a lot more than I normally would, because of the connection aspect. It’s been, it’s been negative in the way of her… almost being addicted to her device, and so there has been struggle at times when I ask for the device back before bed times, and different times if I want her to focus on her homework and she’s not quite ready, however it might be, she will get, she will become challenging with me and not want to give it up so that’s created some struggle for sure during all of this time. So yeah, but it’s definitely changed and I don’t like the amount of time she’s on it, but I allow it based on the situation that we’re in.” [Participant #11, mother] “I would definitely say for our household it's been a negative just because I've noticed…more so, I mean, like they're just fatigued easier. They're more irritable across the board with each other. But I think it's, almost turning into like an addiction. Like my 10-year-old has a really hard time giving up his games. Like, OK, it's time to put it down, like, and there's definitely more fights and definitely more angst in me. Now we have that. ‘Just let me finish this game’. ‘Let me finish this game’. You know, or like my daughter who doesn't create problems but can retreat into her room and just sit and watch, like binge watch TV shows, you know. And so, I would say it's been negative for us just all the way across the board.” [Participant #16, mother]
Theme 4. Parents and children have limited options	“Well, I would honestly not like that much time [screen time] but then I say, well what else can they do? Sometimes I ask them to read and they just read a little and so on, or we go walk the dogs at night, but nothing else.” [Participant #31, mother] “Like [other participant] said, I wish we can take back time, but how things are right now, we do not have a lot to replace it with. Because the sibling that is closest with him in age is 16, 7 years apart, it makes it really challenging for the both of them. She is a good sport and can be but it is really hard. Yeah, I wish it could be different but the circumstances are how they are, just like me drinking a glass every single night is not the greatest thing. That is not for me to change my actions right now because we are in the thick of it. That is kind of where I feel like my youngest is with his screen time and we try to help him and take him off of it. We watch movies together, but there is only so much you can do. There are no alternatives that can be presented to him, I do not have a lot of things to threaten him with. Options are limited.” [Participant #6, mother] “I mean, like I said, he [dad] has really created a schedule for them. And so, he does a good job about saying, ‘nope, right now is a no technology time. Shut it off. Go play together’, or ‘this is outside time’. And so, he's put that together. But there are some days that that doesn't work for his work schedule and he has a meeting at that time. And so that becomes a technology time and it's OK to kind of make those adjustments.” [Participant #19, mother]
Theme 5. ST restrictions	“But now that I have noticed it more, that I have lost more control, and she has been more on the T.V., I have tried to set limits. I tell her, ‘No, no, no, that is enough screen time, we are going to play this’. I try to do things with her, well in my case since she is young, I make her do more manual things. I teach her things, like if I am making tortillas, I have her help me. Although she is little, I want her to interact more with me, that we are together, that she interacts more with me than the television.” [Participant #21, mother] “For me I’ve tried to set certain hours for them, they understand for a few days but then they kind of don't care much about what I tell them. First, I used to tell her, ‘you have certain hours to use the phone, computer, television’, whatever they're using, or I'd tell her to turn off the phone in an hour. She just did it in the first few days and then she would forget and grab it again when she wanted.” [Participant #29, mother] “In my point of view, it is more, much more difficult to restrict it. If you tried to control the personal use that they have, obviously it is very difficult because as I mentioned before, my children talk a lot among themselves and they want to do what other children do. This and especially as I mentioned before they are not interested in doing another activity and they are very clever. My oldest child tells me, ‘I do my homework, I behave well at school, I help you do my chores, why not? Why can't I use it?’ So, it is difficult for me to explain to him that he should do something else. So, it is very difficult.” [Participant #47, mother]
Subtheme 5.1. Children react negatively when ST is restricted	“Yes, we have tried [screen time restrictions] but it's super, super difficult. In truth, my family is very, very difficult. I have seven children, one 18, one 17, one 15, one 13, one 8, and a 3-year-old girl and a 6-month-old baby. It has become very, very difficult for me to set rules on this. Because the children, the two oldest, get very rebellious because they want to be playing with the other children. It’s a fight every day, they say, ‘You don’t let us play, we are not going to the park, we’re going to our friends house to play’. I can’t do anything other than to help their whims that they want to be playing on the computer. You just hear the fighting and shouting here in front of the television. ‘Oh no, they killed me’. Very angry. Oh no, it has been very difficult for me.” [Participant #42, mother] “Like, I want him to, you know, play games with friends and bond because he's so isolated. But my problem is that he feels like he has access 24/7, and my rules, he doesn't listen to my rules. And so, he's going in past bedtime and I'm finding his eyes on screens or playing games and stuff. And I have tried every combination I can think of. So now they're restricted. So now he's more isolated. So, it's hard. It's very hard.” [Participant #10, mother] “It has been bad just because if we don't allow them to, if they ask, ‘can I play on the tablet’ or ‘oh, can I play Xbox?’ and we tell them no, they are at such a young age, they can't really comprehend or know how to calm themselves after that. When we say no, they should, they should kind of be able to calm themselves down. As in like not throw a tantrum once, once we say no. And then we try our hardest not to give in. But at times, if we're trying to cook and…they're just crying and just going like everywhere and not focusing on what we're telling them to do, then we'll just be like, ‘OK, go ahead and go on the tablet. It's fine, but it has to be school related’. It has made their attitude a little bit, in a negative way, just because they're craving to have that screen time when normally we would just go outside. They wouldn't want to go outside.” [Participant #5, mother]
Theme 6. Parents are concerned that their children are not getting enough exercise	“And they have gained weight through the pandemic. I attribute it to all those things...the screen time, the being sedentary, the snacking and all of that stuff. And like...my son used to be you know, he would and my daughter, they would have their basketball or they would have their sports practices every day. And so, it's like not having that motivation. Their normal activities. And they're finally hitting this, like, pre-teen where you're getting all these hormones. So, I'm not necessarily worrying so much about my little ones, but I'm, I am very concerned about my older ones just because that can be detrimental going into your teen years and being overweight and struggling with that and like having a whole year of not doing anything and not having all that, and then not necessarily like me being able to motivate them. I mean, they do PE, so they've been doing yoga for their PE classes. They have to. But I mean, like, it's a 30-minute workout video that they do two days a week, which doesn't really give you any benefit. So, I would say, yes, I am very concerned for my own kids.” [Participant #16, mother] “It worries me because before when they went to school they made them exercise at break time, and now they don't do anything.” [Participant #48, mother] “They’re not doing sports, they’re not running around outside with their friends, yeah, I do have concerns.” [Participant #13, mother]

^a^ST: screen time.

### Theme 1. Total ST has Increased

Parents described how ST changed during the pandemic. ST greatly increased, with children spending much of the day engaged with screens. Screens were used for both school and recreational purposes. The most common nonschool screen activities that children engaged in, as discussed by the parents, were watching television, playing video games (eg, on the Nintendo Switch, Xbox, and app-based games), watching YouTube videos, and spending time on social media platforms such as Facebook, Instagram, and Tik Tok. Several parents remarked that their children played video games often with other people, usually their friends or other family members.

Parents also disclosed the amount of time their children spent on screens for purposes of school vs recreation. These values are presented here to supplement their descriptions. ST increased for families, partly because children attended school remotely; however, parents indicated that their children’s recreational use of screens represented almost half of their total daily ST. Children were on screens an average of 8.21 (SD 2.22) hours a day, with an average of 3.30 (SD 1.59) hours of that time being spent on nonschool activities. While 29% of the sample shared that their children were spending ≤2 hours on their devices for recreational use during the pandemic, 36% of the sample disclosed that their child’s recreational ST was ≥4 hours a day.

### Theme 2. Children are Too Attached to Their Screens

One of the most prevalent themes was that parents felt that their children had become too attached to screens. Many parents used the word “addicted” to describe children’s ST behavior during the pandemic and felt that their children would be on their devices constantly if they were allowed. Some parents described their children’s behavior to consist of waking up and going to bed with screens. Parents noticed an increase in their children’s desire to spend time on screens rather than engage in other activities. A few parents disclosed that their children would even race through meals or family time so that they could return to their screens. Most parents discussed an increase in resistive behaviors (eg, arguing, irritability, and refusal) when children were asked to stop using their devices. Lastly, a few mothers noted that not only the children but also the adults were “addicted” to screens during this time.

### Theme 3. ST has Advantages and Disadvantages but Parents Perceive ST as Mostly Negative

Parents acknowledged both the advantages and disadvantages of ST. Parents recognized the many purposes that technology served during the pandemic, especially by providing an option for their children to continue their education from home. Advantages also included becoming more comfortable with technology, especially for younger users, and providing children, particularly adolescents, with a critical outlet for social interaction. Disadvantages included deprivation of face-to-face social interaction, less physical exercise, concerns about sleep problems, strained eyes, and limited parental oversight. Some parents expressed that it had become more difficult to monitor children’s ST now that their children were spending so much time on screens.

Despite recognizing both the advantages and disadvantages of ST, most parents felt that ST had a more negative rather than positive (or neutral) influence during the pandemic. The effects they observed with regard to ST were manifold and included increased arguments at home, too much attachment to devices, less physical activity, and disobedient behavior. Many parents expressed concern that their children were simply spending too much time on screens and that it had been challenging to enforce ST rules. Some parents described this as a “constant” fight or an ongoing “battle.” A few parents expressed that their children had developed “attitudes” as a result of too much ST. Only 2 parents in the sample indicated that ST was overall more positive during the pandemic.

### Theme 4. Parents and Children Have Limited Options

Many parents discussed how the pandemic greatly limited what children and families could do, and that this subsequently led to an increase in ST. On the one hand, children had to use their screens for school, but they were also no longer able to spend time in their regular activities such as organized sports, attending social gatherings, and connecting with their friends in person. Children missed their friends and felt a sense of connection by being active on the internet. Sometimes this took the form of social media, sometimes playing video games with other children on the internet, and at other times through FaceTime calls with friends or other family members. A lot of the time children felt bored, and the internet provided them with entertainment. A few parents noted that ST offered stress relief for their children and was a way for them to cope during such an uncertain time. Some parents also mentioned that they themselves were out of other options in terms of directly supervising their children while they were working; hence, ST provided their children something to do while they tended to their tasks.

### Theme 5. ST Restrictions

The majority of parents expressed that they had attempted to restrict their child’s ST in some capacity. This was met with varying degrees of success, but many parents reported struggling to enforce their rules. Sometimes parents resorted to taking devices away and hiding them (especially before bed) to keep their children from using them. Some discussed how they ultimately ended up relaxing the rules because they found them to be either unenforceable or that they wanted their children to feel connected to their peers through screens. The limited number of activities was also discussed as a reason for why there was an increase in leniency. A few mothers shared that their ST rules had not changed and continued to be enforced in the same way during the pandemic.

### Subtheme 5.1. Children React Negatively When ST is Restricted

Parents discussed their children’s reactions to ST restrictions and expressed that they tended to respond in negative ways. Parents reported that their children argued with them about pausing their use so they could do something else. For younger children, meltdowns would sometimes ensue, and with both younger and older children, parents reported that they would sometimes ignore their requests to stop using their devices. While most parents reported encountering some type of difficulty or resistance in pausing the use of screens, a few mothers expressed that their children had no problem with stopping ST.

### Theme 6. Parents are Concerned That Their Children are not Getting Enough Exercise

The majority of parents were concerned about their child’s physical activity and felt that they were not getting an adequate amount of physical activity during the pandemic. Some parents noted the absence of organized sports and regular school activities as contributing to the problem. Others expressed that it was challenging to get children outdoors and away from their devices. Virtual physical education (PE) was indicated by some parents, but this was mostly dismissed as inadequate in part because it was offered too infrequently, or in some cases cameras were not required to be turned on during the sessions; thus, children could easily opt out of participating. A few parents expressed that their children were not getting any exercise at all during this time.

## Discussion

### Principal Findings

The pandemic has upended families’ regular routines, and an increase in ST has been one consequence. This study provides insight into how families have been navigating ST during the pandemic. Parents expressed concerns about total ST, the addictive nature of it, and the lack of physical activity. One prepandemic study found that 45% of teenagers reported that they were always connected to the internet [11]; this percentage is likely to be much higher now and to factor in younger children as well. Similar to a prepandemic study that reported that some parents of preschoolers described their child as being “addicted” to ST [26], the parents of children of varying ages in this study were concerned about the addictive nature of screens. Several parents described addictive behaviors that are consistent with symptoms of problematic internet usage, including preoccupation with being on the internet and withdrawal symptoms [27,28].

How children are using screens and what content they are viewing are critical for advancement of the understanding of how ST influences child development. A significant portion of ST has been spent engaged in remote-schooling during the pandemic, but parents also expressed that a considerable proportion of ST has been spent in recreational use. More than one-third of the sample indicated that their child’s recreational ST accounted for ≥4 hours per day. Some parents also linked their child’s ST to concerns regarding the lack of physical activity. Increased ST in a country where obesity is already an epidemic, with 18.5% of the youth considered to have obesity before the pandemic [29], may have a profound lasting effect on weight gain in children.

Some parents expressed that their children were engaged in a PE course that was delivered remotely by their child’s school. Nonetheless, parents were not very optimistic about children gleaning many benefits from the classes in part because they were offered too infrequently or because their children did not participate. A few parents also discussed the potential engagement of their children in games on their devices, which include movement; it will be helpful for future studies to assess sedentary vs nonsedentary game activities and quantify the benefits accrued from participating in screen-based physical activity games. Studies have reported that some active video games are effective in increasing physical activity [30], but more studies are needed in this area. In a recent publication, promoting physical activity via screens was recommended as a healthy way to engage in ST during the pandemic [31]. How these recommendations have been (and will continue to be) translated into practice will be important to assess. Unfortunately, most parents in our study did not report activity-based screen games as regular activities their children engaged in when using their screens, and most parents were worried about the amount of physical activity their children were getting. Additionally, 4 of 5 households have some type of device that can be used to play video games [32], and 75% of US households include a minimum of 1 person who plays video games [33], a number that is likely to increase as some families in this study expressed that they purchased a gaming device for entertainment purposes during the pandemic, which has ramifications for continued long-term use.

Parents indicated that engaging in social media was an acceptable form of ST, especially because it provided their children an opportunity to connect with others during the pandemic. Findings on the effects of social media on development are mixed, with some studies showing an association between engagement on social media and protection against depression [12] and little to no impact on social skills [34], while another study reported that interacting on social media (specifically Facebook) is related to lower levels of well-being among young adults [35]. One report describing 3 time-use-diary studies in 3 countries found little support for the association between digital engagement and lower levels of adolescent well-being [36]. Further studies are needed to examine how children are connecting with others through screens, as well as the frequency, to determine how this relates to maintaining feelings of connectedness during lockdowns and periods in which face-to-face interactions with one’s peers are greatly reduced or even nonexistent. Additionally, the AAP recommends that parents coview media with younger children, but this has become more challenging for many families as parents attempt to balance their own work (possibly from home) and family life during the pandemic. Updated guidelines and strategies by the AAP may be helpful for parents in navigating screen use in the current situation, and in potentially similar situations in the future, considering the cyclical nature of the lockdowns and unpredictability of SARS-CoV-2.

### Limitations and Strengths

Our findings should be interpreted with caution. Parents in our study reported that their children were spending 8 hours a day on screens. This average is not very different from the finding of a 2010 Henry Kaiser Foundation report that revealed a daily average of 7 hours 38 minutes of media use in 8- to 18-year-olds [37]. A noticeable discrepancy is that the >7 hours in the Kaiser report represented entertainment media and was reported by youth aged ≥8 years, while the average in this study was reported by parents and represented a combination of education and entertainment media for children aged ≥5 years. This gap should be examined in more detail to determine the nature of the difference. Additionally, we did not focus on 1 specific age group, which would be helpful in gaining a more in-depth understanding of how the pandemic is affecting children by age. However, this was by design. Most parents in our study had more than 1 child (average 3), and our goal was to capture what was happening within the family unit.

The strengths of this study include a sample in which all families shared the same unique situation of children attending school remotely. In addition, parents were invited to participate in the focus group sessions 9-11 months into the pandemic, which offers a clearer insight into how families have adapted and settled into new routines around ST since the pandemic initially began. However, this study serves as a starting point, and further studies are needed to provide cross-sectional insights into how the pandemic has influenced ST in families.

A few parents shared how their own ST had increased, and that it disrupted them from being present with their children or other family members, which left them feeling guilty. Parental ST use during the pandemic was not explored in-depth in this study but is an area that warrants further attention. This may be of special interest to parents of young children because research suggests that when mothers used their devices around young children, they were less likely to talk to them [38]. However, many parents are spending unprecedented amounts of time with their children and the total amount (and quality) of conversation may be sufficient over the course of the day. The emotional connections that parents are making with their children is of particular importance with regard to child health [39], as well as the quality of parenting and ST [40], and studies should examine how parents are connecting with their children during the pandemic. Additionally, more than half of our participants were essential workers, or lived with one, more than one-third being working mothers, and studies have reported that both essential workers [7] and working mothers [41,42] have been disproportionately affected during the pandemic. Future comparative studies should target parents across different socioeconomic statuses, as well as essential workers and working mothers, to determine whether differences exist between their levels of ST. The impact of ST on long-term development needs to be examined through a new lens because families have never been in this particular situation before. Although there are risks associated with ST, there are also benefits, and whether the positives outweigh the negatives or vice versa during a pandemic is yet to be determined.

### Conclusions

ST has become even more ubiquitous in the home setting, and 2 generations of children will have spent an unprecedented amount of time on screens and social media over the past year. ST and social media are a relatively nascent force, and one that is transforming rapidly; therefore, we do not yet know the panoply of long-term effects that may be associated with its use, especially during a pandemic where there has been digital reliance to maintain a semblance of normalcy. For very young children, the pandemic has occurred during a time when healthy habits first begin to be established. Health care professionals, child psychologists, and developmental psychologists may want to focus on preventing potential negative long-term sequelae of heavy ST, which may include mental, socioemotional, and physical health challenges. The goal of this study was to describe families’ experiences of using screens during this time, and to inform future studies. It is important that future studies examine the long-term effects of excessive ST, and preemptively introduce ways to redirect children’s ST habits as the country attempts to establish a new normal.

## Abbreviations

AAP: American Association of Pediatrics
PE: physical education
ST: screen time
